# Role of Self-Assembled Surface Functionalization on Nucleation Kinetics and Oriented Crystallization of a Small-Molecule Drug: Batch and Thin-Film Growth of Aspirin as a Case Study

**DOI:** 10.1021/acsami.1c00460

**Published:** 2021-03-24

**Authors:** Fiora Artusio, Francesco Fumagalli, Andrea Valsesia, Giacomo Ceccone, Roberto Pisano

**Affiliations:** † Department of Applied Science and Technology, Politecnico di Torino, corso Duca degli Abruzzi 24, 10129 Torino, Italy; ‡ European Commission, Joint Research Centre (JRC), via E. Fermi 2749, 21027 Ispra, Italy

**Keywords:** crystallization, functionalization, aspirin, SAM, thin film

## Abstract

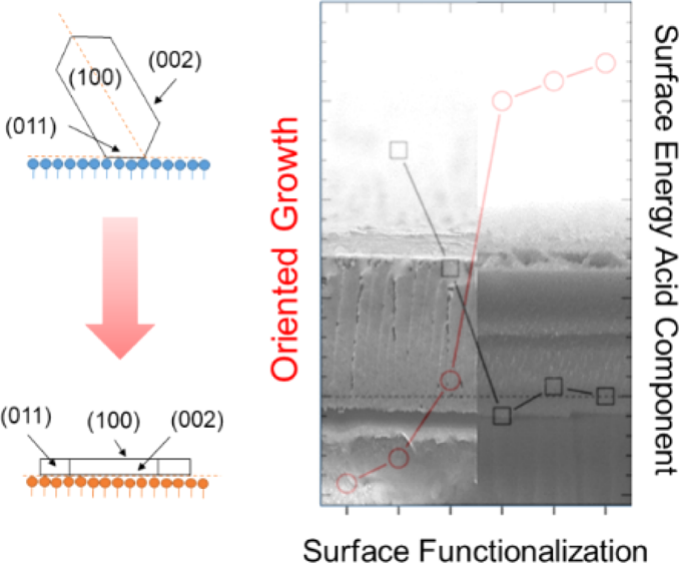

The present paper assesses the heterogeneous nucleation of a small-molecule drug and its relationship with the surface chemistry of engineered heteronucleants. The nucleation of aspirin (ASA) was tuned by different functional groups exposed by self-assembled monolayers (SAMs) immobilized on glass surfaces. Smooth topographies and defect-free surface modification allowed the deconvolution of chemical and topographical effects on nucleation. The nucleation induction time of ASA in batch crystallization was mostly enhanced by methacrylate and amino groups, whereas it was repressed by thiol groups. In this perspective, we also present a novel strategy for the evaluation of surface–drug interactions by confining drug crystallization to thin films and studying the preferential growth of crystal planes on different surfaces. Crystallization by spin coating improved the study of oriented crystallization, enabling reproducible sample preparation, minimal amounts of drug required, and short processing time. Overall, the acid surface tension of SAMs dictated the nucleation kinetics and the extent of relative growth of the ASA crystal planes. Moreover, the face-selective action of monolayers was investigated by force spectroscopy and attributed to the preferential interaction of exposed groups with the (100) crystal plane of ASA.

## 1 Introduction

The industrial manufacturing of drugs often involves crystallization steps for the isolation, purification, or delivery of active pharmaceutical ingredients (APIs).^1^ Being one of the most widespread unit operations, crystallization nowadays represents a relevant percentage of the drug manufacturing process in terms of time and cost. Crystallization not only opens access to an easy-to-handle and stable product but also strongly affects the final product properties, such as flowability, biological activity, and tableting.^2^ Such features are directly correlated with the crystal form, habit, and size, which result from the crystallization step. Many different approaches have been recently proposed to achieve a higher degree of control over the process and ensure the meeting of strict pharmaceutical quality constraints. Among these, surface-induced crystallization represents a valuable tool for crystal engineering. The crystallization pathway can be modified by tailored heteronucleants without altering the operating conditions of the process, that is, pH, temperature, or solvents. Polymorph selection, crystallization confinement, crystal size, and density control are just a few examples of the application of such a technique.^3^

In the framework of surface-induced crystallization, polymeric, silica, or gold substrates have been widely applied for promoting and directing the crystallization of pharmaceuticals and biopharmaceuticals.^3^ Various surface properties, such as morphology, charge, chemical composition, or crystalline order, can be exploited to tune the crystallization pathway of target molecules. The surface–solute interaction may involve just the surface of heteronucleants, as for films or full particles, or also the bulk of the material, as for porous structures. The former can be selected to study epitaxial phenomena,^4^ secondary interactions between the substrate and solute,^5^ or the effect of charge distributions.^6,7^ The latter relies on confinement to boost nucleation kinetics of APIs with polymeric gels^8,9^ or promote protein crystallization with the help of agarose gels and mesoporous structures.^10,11^ However, little attention has been paid to the deconvolution of topographical and chemical effects induced by surfaces on nucleation, often leading to the difficult interpretation of results. The isolation of the two components is desirable to rigorously understand the role of surface–API interactions during crystallization, and it may be achieved with smooth surfaces exposing different chemical groups.^12^

In this perspective, self-assembled monolayers (SAMs) have the potential to be applied as heteronucleants because they provide a reliable and reproducible method to tailor surface chemistry allowing precise control of the physicochemical properties of heteronucleants.^13^ In our previous study, we proposed a synthesis protocol for SAMs, which ensured a robust functionalization of glass with selected groups and roughness below 0.15 nm.^14^ SAMs are the result of a spontaneous organizational process and provide a versatile platform for studying self-organization, interfacial phenomena, and the competitive interactions occurring among surface, solute, and solvent molecules.^15^ Many applications of SAMs in biotechnology,^16^ bio-sensoring,^17^ organic electronics,^18^ and photonics^19^ have been reported. With regard to crystallization, the self-assembly of selected building blocks in monolayers or multilayers has been adopted to create supports for polymorph selection^20^ and protein crystallization,^21^ as well as for oriented growth^22^ and nucleation kinetics.^23^ For example, SAMs were patterned to create hydrophobic and hydrophilic areas to force the crystallization of glycine at the nanoscale^24^ or even coupled to porous layers, such as metal–organic frameworks, for crystal engineering.^25^

From the perspective of the pharmaceutical crystallization process, most studies involving heteronucleants have been carried out in batch.^3^ As reported in Table 1, batch crystallization involves macro-volumes of drug solution and long onset times. In addition, many experiments need to be performed to get a statistically significant dataset, especially when heteronucleants are involved. In this scenario, the confinement of API crystallization to thin films guarantees considerable savings in terms of time and amount of API. Spin-coating crystallization (SCC) is driven by solvent evaporation, which is responsible for creating the supersaturation conditions and, hence, the driving force for nucleation. However, because of continuous solvent removal, the exact supersaturation level that triggers nucleation is unknown. Nevertheless, precise control over film thickness can easily be achieved. Moreover, trials involve minimum amounts of API, allowing crystallization of highly soluble drugs even in single-component solvents. The avoidance of local gradients of API concentration that may affect static batch crystallization can also be avoided, thus guaranteeing isotropic interactions between API and heteronucleants during the crystallization process (see Figure S1). Regarding the testing time, SCC is completed within a few minutes, whereas batch processes require many hours or even months. SCC also facilitates successive X-ray diffractometry (XRD) crystallographic and orientational studies, as no preliminary treatments are required. Conversely, samples obtained by batch crystallization accounts for rinsing and drying steps to remove residual solvents or non-specific crystals,^12^ which could potentially alter XRD analyses.

**Table 1:** Comparison between Batch Crystallization by Cooling and SCC Applied to the Lab-Scale Study of Heterogeneous Nucleation

	batch	SCC
achievement of supersaturation	cooling	solvent evaporation
supersaturation	defined	unknown
volumes of the API solution	μL (hundreds) to mL	≤100 μL
amount of drug required	mg to g	μg
duration of the experiment	few hours up to months	≤5 min
study in single-component solvents	depends on drug cost and solubility	yes
API–surface interaction	anisotropic	isotropic
post-treatment for XRD studies	rinsing and drying	none

In recent years, several studies on the confinement of pharmaceutical crystallization to thin films have been reported in the literature. The ability of selected substrates to modify the structural order of materials near the interface was exploited to study thin-film phases.^26^ Thin films of pharmaceuticals were prepared to enhance the drug solubility and dissolution rate,^27^ discover new polymorphs,^28,29^ control the nucleation of specific crystal forms and stabilize amorphous forms,^30^ alter the texture and form of crystals by coupling them with thermal treatments,^31^ study tautomerism,^32^ and the crystallization behavior of drugs in different solvents^33^ and with polymeric additives.^34^ For example, aspirin (ASA) deposited on oriented pyrolytic graphite by spin coating led to dimer rods reflecting the underlying pattern as a result of nonpolar interactions.^35^ Moreover, metastable forms of acetaminophen were stabilized when the spin coating was followed by thermal treatments^36^ or coupled to polymeric surfaces.^37^ Thin composite layers of drug and matrix materials have also been proposed as a platform for drug delivery,^38,39^ in alternative to nanoparticles.^40,41^ The use of thin films has also been proposed for the continuous manufacturing of drugs.^42^

In this paper, we discuss the use of surfaces coupling chemical modification to sub-nanometer-scale roughness to study the crystallization of a model drug. We synthesized SAMs on glass supports and used them to assess the effect of controlled superficial chemistries on the nucleation induction time and the preferential growth of ASA. First, we employed batch crystallization to evaluate the nucleation time of ASA on various SAMs and quantify their inducing or inhibiting action. Then, we proposed SCC as a tool to evaluate the surface–drug interactions by confining ASA crystallization to thin films and studying the preferential growth of ASA crystal faces imposed by SAMs. The results that emerged from batch and SCC were finally confirmed by quantifying the adhesion force between selected chemical groups and the ASA (100) crystal plane.

## 2 Materials and Methods

**Figure 1:**
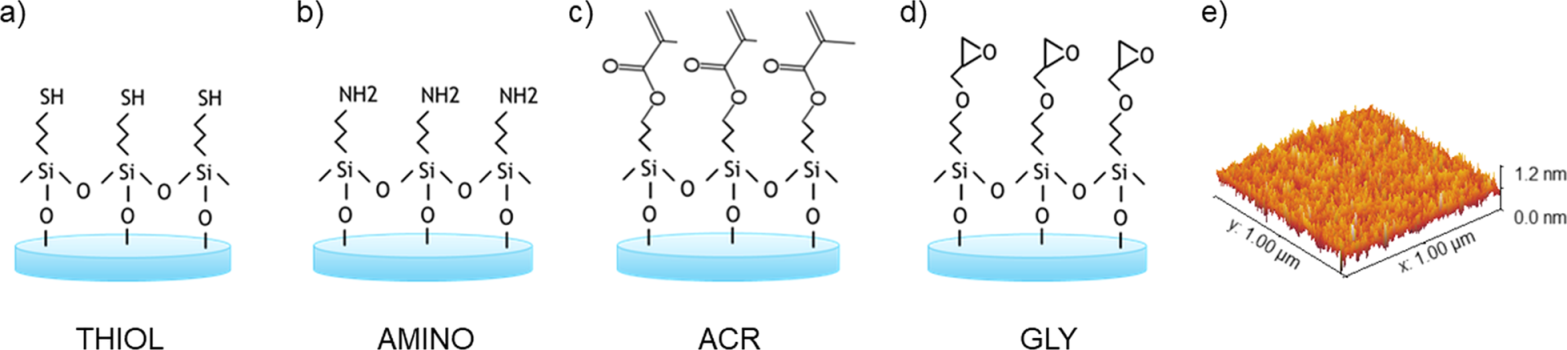
Overview of the surface chemistries employed in the present study: (a) THIOL, (b) AMINO, (c) ACR, and (d) GLY SAMs. (e) Representative topography of a glass surface that has been functionalized with THIOL SAM.

The optimized synthesis of SAM-functionalized surfaces is described in our previous study^14^ and in the Supporting Information. Briefly, glass coverslips were pre-activated by piranha solution, rinsed, and transferred into 0.054 M silane solutions in anhydrous toluene for max. 15 h to achieve SAM grafting. In this study, we used the following silane molecules: 3-aminopropyltrimethoxysilane (AMINO), 3-glycidyloxypropyltrimethoxysilane (GLY), 3-mercaptopropyltrimethoxysilane (THIOL), and 3-(trimethoxysilyl)propylmethacrylate (ACR), see Figure 1. For simplicity, we will refer to the respective SAMs as “AMINO”, “GLY”, “THIOL”, and “ACR” SAMs, respectively. Characterization details are also reported elsewhere^14^ and additional information can be found in the Supporting Information. Three probing liquids (H_2_O, glycerol, diiodomethane) were used for contact angle analyses according to van Oss–Chaudhury–Good (vOCG) model for the dispersive, acid, and base surface tension components, γ^LW^, γ^+^, and γ^–^. Topography was recorded via atomic force microscopy (AFM) in tapping mode using Si_3_N_4_ cantilevers with a scanning frequency of 0.8 Hz and 1 × 1 μm^2^ analysis area (256 lines). The nucleation kinetics of ASA was studied in 24-well plates. ASA was dissolved in an ethanol/water mixture (38/62 v/v) and filtered at 0.22 μm. The starting concentration was 31.6 mg/mL and the temperature was set at 15 °C to favor heterogeneous nucleation. SAMs with 125 μL of ASA solution were placed in each well covered with a lid. Each plate was placed inside a temperature-controlled chamber fluxed with dry N_2_. The chamber was designed to inspect the wells via time-lapse transmission stereomicroscopy.

**Figure 2:**
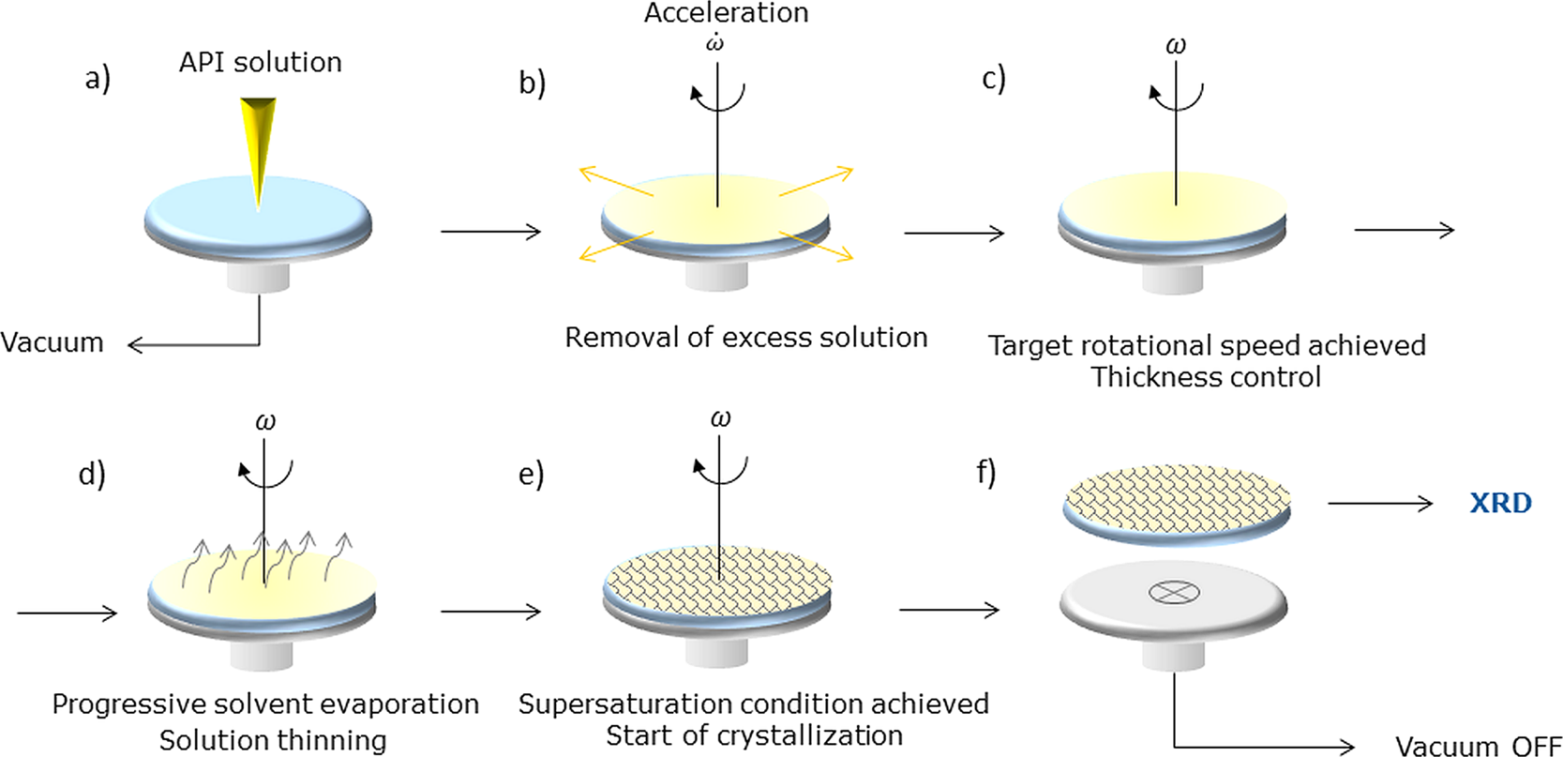
Schematic representation of SCC of APIs: (a) solution casting, (b) initial acceleration, (c) constant rotational speed and increasing supersaturation, (d) start of crystallization, (e) end of crystallization, and (f) sample removal.

ASA thin-film crystallization was achieved via a spin coater using filtered ASA/ethanol 50 mg/mL solutions. ASA solution (100 μL) was pipetted onto the SAM-functionalized substrate. Spinning parameters were spin time 5 min, acceleration 500 rpm/s, and rotational speed varied between 500 and 4000 rpm to control thickness. Solvent progressive evaporation induced supersaturation conditions and ASA nucleation, followed by fast crystal growth. An overview of SCC is given in Figure 2. Variable angle ellipsometry (65, 70, and 75°) was employed to measure the ASA film thickness using a Cauchy model. Field emission scanning electron microscopy (FE-SEM) was used to investigate the morphology and thickness of ASA thin films. Sample cross-sections were sputtered with a thin gold layer. The accelerating voltage was 2 keV, a through-lens detector was used, and the working distance was set at 3.1 mm. ASA thin films were analyzed with no preliminary treatments with an X-ray diffractometer operated in the Bragg–Brentano mode (X-ray lamp, *I* = 40 mA and *V* = 40 kV). A Göbel mirror, a 2.5° soller, and a 0.3 mm pinhole were inserted along the primary beam path. A 0.6 mm slit and a 2.5° soller were mounted on the secondary beam path. 2θ ranged from 6 to 35°, the step size was 0.02°, and the time per step was 15 s.

The interaction between ASA (100) crystal face and selected silane chemistries was evaluated with AFM through force spectroscopy. The experiments were performed in a clean room. Si_3_N_4_ tips were functionalized with silanes following a similar procedure as for glasses^14^ and then used to collect force–distance curves. Each probe was preliminarily calibrated using silicon wafers, and the spring constant was calculated. A UV-cleaned tip was taken as a reference. Force–distance curves were measured using an ASA crystal grown in bulk using batch crystallization. The crystal was placed on the AFM stage with the extended (100) face facing the tip. Each measurement was repeated at least on 15 different spots of the ASA crystal surface.

## 3 Results and Discussion

### 3.1 Batch Crystallization of ASA

Different chemistries immobilized on glass surfaces were selected to carry out ASA crystallization in batch trials. The optimized synthesis of supports has been presented in our previous publication, where the surface attributes of SAMs were thoroughly characterized.^14^ A schematic of the investigated SAMs, which exposed thiol, amino, methacrylate, and glycidyloxy groups, is sketched in Figure 1a–d. Defined and reproducible physicochemical surface properties, such as roughness and surface coverage, were obtained by functionalizing glass with monolayers of silanes. Additional details on surface characterization by X-ray photoelectron spectroscopy, contact angle, and SEM are reported in the Supporting Information (see Tables S1–S3 and Figure S2). The selected functionalizing agents were grafted to the surface via condensation reactions, and all had the same number and type of head groups, that is, three methoxy groups, as well as the same hydrocarbon spacer length, that is, three carbon atoms. Particular attention was paid to the preservation of pristine glass topography after the functionalization with SAMs. This aspect is particularly important since surface discontinuities and roughness can act as nucleation promoters, thus masking surface chemistry effects. As reported in Figure 1e, SAM grafting did not alter glass surface topography, as RMS roughness was always below 0.15 nm, both for pristine and functionalized glasses.^14^

In order to relate the different surface chemistries to the crystallization of ASA, a series of physicochemical surface properties of SAM-functionalized substrates was investigated, such as the surface zeta potential (SZP),^14^ the surface tension and its components, and the number of hydrogen bond (HB) donor/acceptor groups, which are reported in Table 2. SAM chemistry was specifically designed in order not to introduce excessive variations in the overall surface tension γ but to play with its components by varying the exposed group. Surface tension components were calculated according to the vOCG model^43^ starting from contact angles reported in the Supporting Information (Table S1); Lifshitz–van der Waals (γ^LW^), acid (γ^+^), base (γ^–^), and polar (γ^AB^) components were identified. The presence of the propylic chain and end group of SAMs did not significantly alter the dispersive interactions, as all the samples resulted in comparable γ^LW^ components. It has to be noticed that SAMs exposing amino groups showed a slightly higher γ^LW^, which in turn affected the overall surface tension, making it the highest among the investigated SAMs. When exposed to the atmosphere, NH_2_ groups are prone to attract charged particles, leading to increased γ and γ^LW^ because of surface contamination by hydrocarbons and carbonyls.^14^ With regard to the polar components, methacrylate groups resulted in the highest acid contribution, whereas amino groups displayed the largest value of the basic surface tension component. Regarding SZP, THIOL and AMINO SAMs displayed the largest negative (−42.3 mV) and positive (+14.9 mV) values, respectively. GLY and ACR SAMs, instead, had approximately the same SZP (−24.7 and −21.3 mV, respectively). Finally, both THIOL and AMINO SAMs had one HB donor and one acceptor group, whereas ACR and GLY SAMs only had two donor groups. The determination of such surface properties will help in the understanding of the molecular interactions between surface and API.

**Table 2:** Surface Tension and Its Dispersive, Acid, Base, and Polar Components and the Number of Hydrogen Bond Donor (HBD) and Hydrogen Bond Acceptor (HBA) Groups of Activated Glass and SAMs

	γ, mJ/m^2^	γ^LW^, mJ/m^2^	γ^+^, mJ/m^2^	γ^–^, mJ/m^2^	γ^AB^, mJ/m^2^	HBD	HBA
activated glass	63.6	38.6	3.5	44.8	25.0	1	1
THIOL SAM	43.0	36.3	0.8	13.7	6.7	1	1
AMINO SAM	56.0	43.2	1.1	36.8	12.8	1	1
ACR SAM	47.6	38.6	2.3	8.7	8.9	2	0
GLY SAM	44.9	37.3	1.0	15.0	7.6	2	0

The interaction of SAMs with a model drug molecule, namely, ASA, was analyzed in terms of nucleation kinetics and thermodynamics. ASA was selected as it is representative of small organic compounds and is commonly used as a model in pharmaceutical crystallization.^44,45^ Batch crystallization of ASA over SAMs was carried out in static conditions by cooling ethanol/water mixtures to 15 °C so as to achieve supersaturation *S* = 1.8. The selected *S* resulted from a compromise, which ensured conditions for studying heterogeneous nucleation. On the one hand, too high supersaturation (*S* > 2.5) promoted the homogeneous nucleation of ASA crystals in bulk, preventing the study of surface effects. On the other hand, as supersaturation represents the driving force of crystallization, too low supersaturation (*S* < 1.6) hindered nucleation, and the observation of the first crystals could require an impractically long period of time.

**Figure 3:**
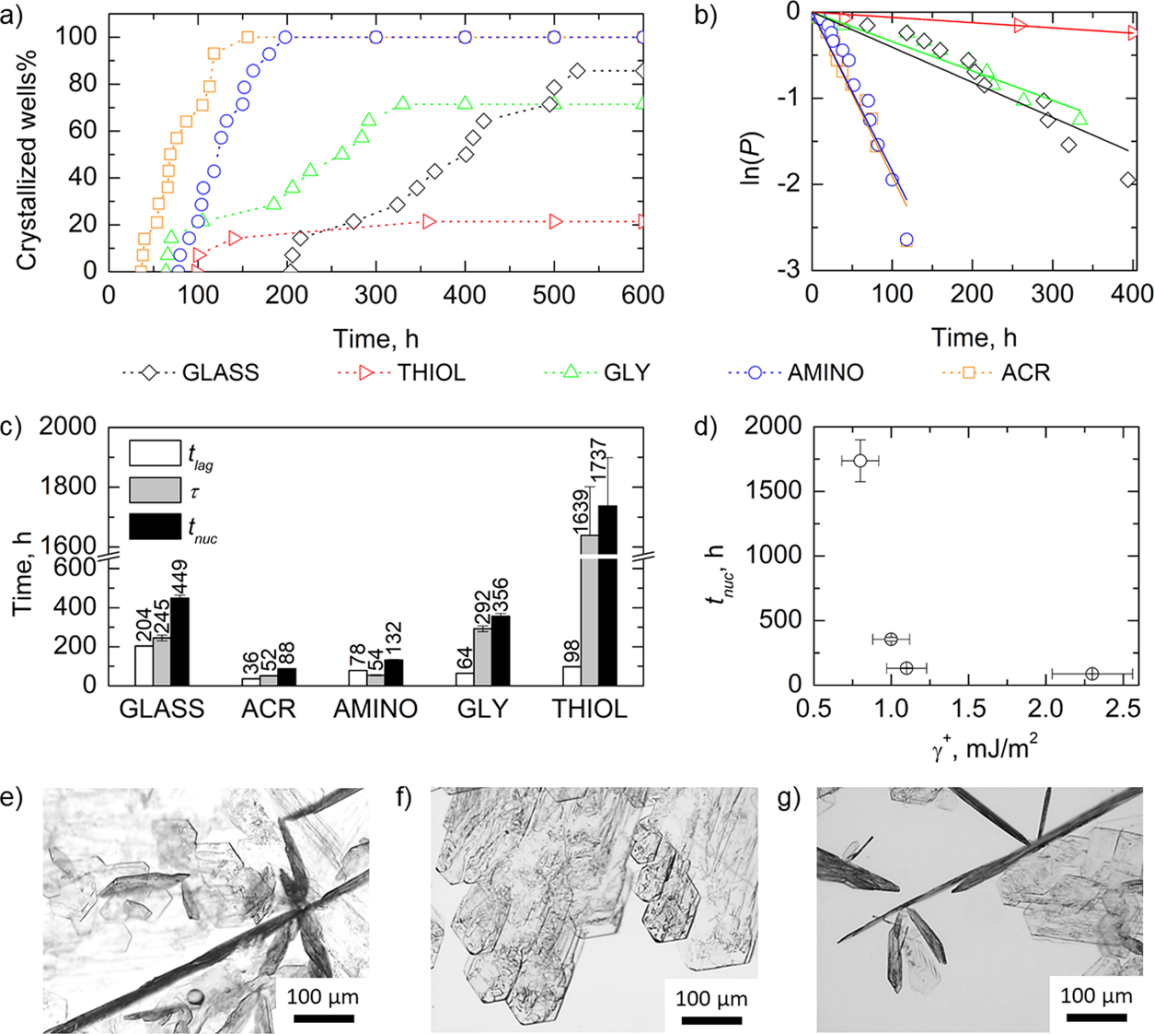
(a) Percentages of crystallized wells as a function of time for untreated glass and SAMs carrying methacrylate (ACR), amino (AMINO), glycidyloxy (GLY), and thiol (THIOL) groups. (b) Linear fitting of experimental data with Poisson’s law for the calculation of τ. The corresponding lag times were subtracted from the kinetic data to set all the onset of the curves to zero. (c) *t*_lag_, τ, and *t*_nuc_ obtained on different surfaces with batch crystallization. (d) *t*_nuc_ vs γ^+^ of the corresponding SAMs. Error bars correspond to standard deviations. Representative optical microscope images of ASA crystals grown on (e) glass, (f) ACR, and (g) THIOL SAMs.

The probability of interaction between API molecules and SAMs was enhanced by maximizing the ratio between the exposed interface and the API solution volume. Such a condition was accomplished by using multi-well plates and minimizing the volume of API solution. The low level of liquid in each well ensured a high ratio between the interface with SAMs and the solution volume, increasing the probability of observing surface-induced crystallization. At the same time, SAMs were completely covered with a thin layer of liquid. Each well was inspected by time-lapse optical microscopy for the appearance of the first crystals. The time needed for nucleation by far exceeded the time needed by nuclei to grow to a detectable size. Therefore, the detection of the first crystals could be considered as the nucleation time. In this way, the cumulative distribution of the probability of encountering nucleation events in wells containing different SAMs was calculated, as sketched in Figure 3a. Glass was taken as a reference surface to compare crystallization outcomes. Wells showing immediate crystallization just after the cooling step were excluded from the statistical data analysis because their fast and uncontrolled nucleation was likely due to the presence of impurities. As can be seen from the graph, all the experiments were characterized by an initial lag time, *t*_lag_, which was related to the time required for ASA molecules to diffuse toward the surface, organize themselves into clusters, and finally stabilize into nuclei. The onset and completion of nucleation events followed. The initial lag time was attributed to the extremely low surface roughness of SAMs since it has not been reported when porous supports, polymers, or rough surfaces were used as potent heteronucleants to catalyze nucleation.^12,46^ The absence of superficial asperities or discontinuities repressed nucleation kinetics but allowed for the isolation of chemical effects.

Different trends were observed according to the surface chemistry of heteronucleants. An indication of the heterogeneous nucleation events outpacing the homogeneous ones emerged from the different percentages of crystallized wells obtained at the end of the experiment (600 h). This observation was taken as an indicator of homogeneous phenomena not proceeding at appreciable rates. Hundred percent of wells including ACR and AMINO SAMs nucleated within 200 h, whereas glass and GLY SAMs only led to 70–90% of crystallized wells. On the opposite side, successful crystallization on THIOL SAMs was observed only in 20% of the experiments. After the determination and subtraction of the corresponding *t*_lag_, the onset of the curves was set at *t* = 0, and data were fitted with Poisson’s statistical law (*R*^2^ > 0.95), as depicted in Figure 3b. The slope of the data linear fit (see eq 4 in Supporting Information) corresponded to the nucleation induction time, τ. Then, the overall nucleation induction time, *t*_nuc_, defined as
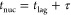
1was calculated. The values are reported in Figure 3c.

Among all the tested conditions, ACR SAM was found to be the most nucleation-promoting surface, leading to the lowest *t*_lag_ and τ and to 100% crystallization success. The appearance of the first ASA crystals in solution required less than 90 h, which corresponded to a 5-fold enhancement of nucleation kinetics compared to untreated glass. AMINO SAM was slightly less performing, with 50% higher *t*_lag_ but still 100% probability of observing nucleation events. The induction of ASA nucleation promoted by the two surfaces denoted their affinity toward the API. With regard to ACR SAMs, we hypothesized that the formation of favorable HBs between surface carbonyl groups and ASA carboxyl groups could help nucleation. ASA dimeric synthon involves intra-molecular HB between −OH/–COOH and two −COOH groups.^47,48^ We hypothesized that the interaction with the superficial methacrylate groups could favor its formation because of molecular mimicking. Overall, ACR SAMs were extremely active in reducing the entropic penalty required for ASA nucleation. With regard to amino groups, they had a marked basic characteristic, as confirmed by high γ^–^ component and positive SZP. ASA molecules are known to act as weak acids in solution. AMINO SAMs could promote favorable acid–base interactions with the solute molecules, reducing the time needed to observe crystals. A different behavior was observed for GLY SAMs since ASA nucleation kinetics was characterized by short *t*_lag_ but long τ and lower probability of successful crystallization. More specifically, the slightly negative value of SZP could encourage the initial diffusion of ASA toward the surface, as previously observed for ACR groups, and thus be beneficial for shortening *t*_lag_. However, the lack of acid–base or mimicking effects slowed down the nucleation kinetics, limiting the interaction to HB with surface ether groups, which turned out to be a less effective mechanism. At the opposite end, THIOL SAMs were extremely active in inhibiting ASA nucleation. τ was approximately 4-fold longer, and the probability of encountering nucleation events was extremely low. The nucleation inhibition observed for THIOL SAMs could be mainly attributed to the strong negative potential of the no-slip plane over it. The accumulation of charged ions in this region may hinder the interaction of ASA molecules with the thiol groups and impede the beneficial reduction of the nucleation free energy barrier provided by the surface.

A macroscopic surface property was related to the action of SAMs toward ASA nucleation, as sketched in Figure 3d. The acid component of surface free energy γ^+^ as derived from the vOCG model describes the ability of a surface to interact with a basic (or electron density donor) surface through polar interactions (dipole–dipole and hydrogen bonding). Increasing γ^+^ enhanced nucleation kinetics, and the SAM inducing ability was saturated for γ^+^ > 1 mJ/m^2^. From an atomic point of view, the exposed surface chemistry of SAMs had a dramatic impact on the nucleation kinetics of ASA because of different mechanisms of interaction. Therefore, by engineering surface properties such as surface tension components, zeta potential, and exposed chemical groups, it was possible to induce a controlled acceleration or repression of nucleation kinetics (relative to nucleation onto the uncoated glass). SAM chemistry, however, did not affect the ASA crystal form or habit since platelet-like monoclinic crystals were observed on all the surfaces, as shown for glass, ACR, and THIOL SAM in Figure 3e–g. Additional optical micrographs are reported in Figure S3.

### 3.2 Crystallization of ASA in Thin Films

The assessment of the impact of surfaces on ASA batch crystallization pointed out a marked relationship between SAM chemistry and API. In this framework, we crystallized ASA as thin films over SAMs to get further insights into the phenomena occurring at the interface. The confinement of drug crystallization to thin films through spin coating techniques could support the mechanistic understanding of surface–API interactions. SCC carried out on SAMs guarantees an isotropic interaction between API and surface. During the process, all the SAM end groups are equally accessible by the drug molecules since the solution thickness is constant over the surface and precisely controlled by spinning. The presence of local gradients of API concentration that may affect static batch crystallization could thus be limited. Besides, SCC may represent a powerful tool for crystallization studies since extremely small thicknesses may be achieved, thus minimizing the probability of encountering impurities that could potentially act as nucleation sites.

In the present study, SCC was applied to ASA crystallization in ethanol following the procedure highlighted in Figure 2. The interplay among solute–solvent, solute–surface, and solvent–surface interactions can have a strong impact on nucleation.^49^ In this scenario, the ethanolic mixture used for batch crystallization was substituted by a single-component solvent to avoid complex interactions between API and solvents mixtures and focus on the interaction between the surface and the solute. Moreover, if ethanol/water mixtures were used, a nonuniform film would be obtained during SCC because of the difference in volatility between the two liquids. Consequently, zones with higher or lower supersaturation according to the local evaporation rates would be formed, resulting in nonhomogeneous ASA crystallization. Dealing with single-component solvents ensured precise control over crystallization conditions, using a limited amount of API. The thickness of the crystallized thin film was precisely controlled by acting on a rotational speed, ω, as the centrifugal force mainly dictates the amount of solution to be retained over the surface. The thickness of ASA thin films crystallized on THIOL SAMs measured by ellipsometry and FE-SEM as a function of ω is reported in Figure S4. Thickness was about 3.6 μm for ω = 500 rpm and progressively decreased while increasing the rotational speed. A plateau around 180 nm was finally reached for ω > 1500 rpm.

**Figure 4:**
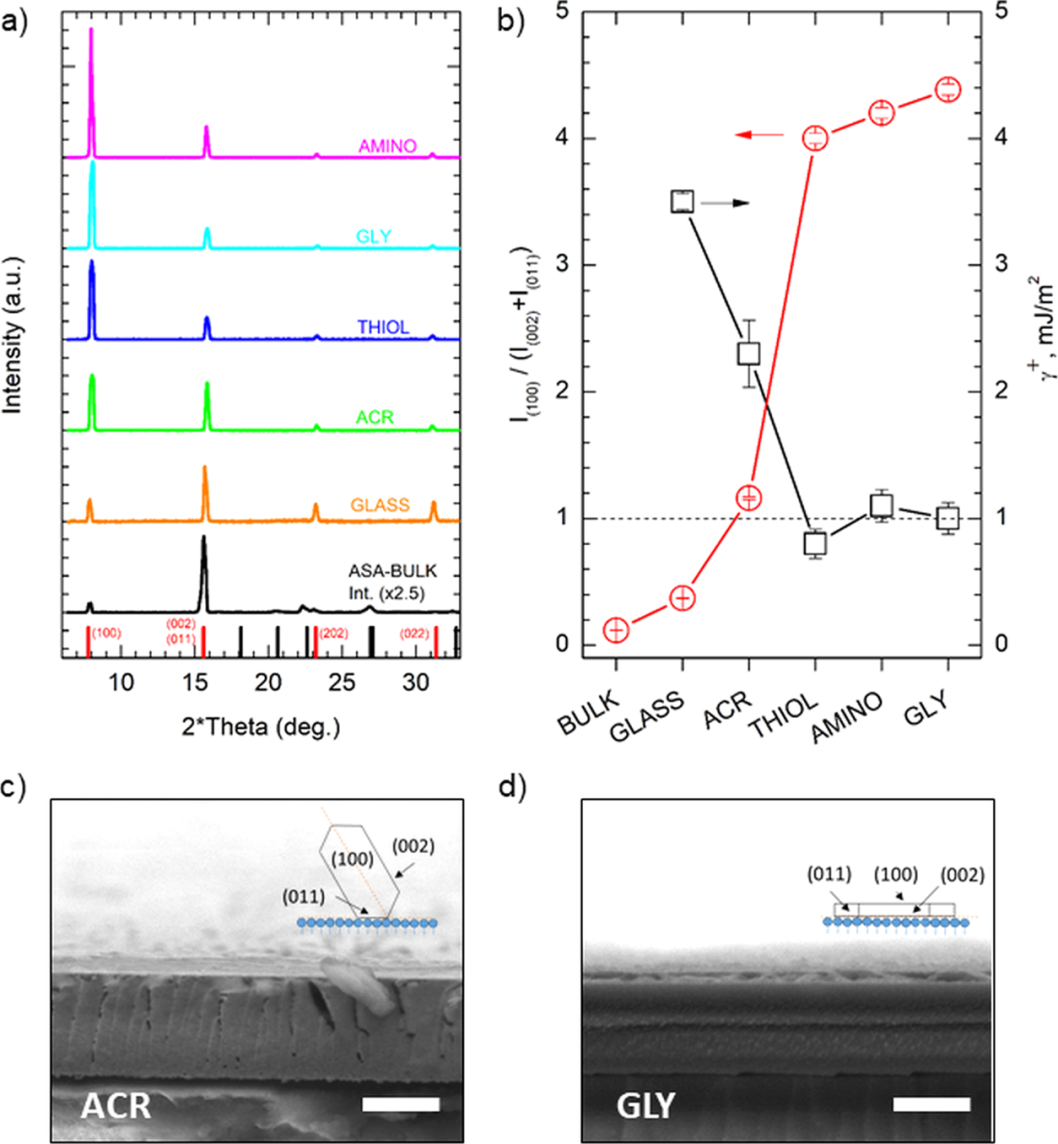
(a) XRD diffractograms of ASA thin films crystallized on (from up to down) AMINO, GLY, THIOL, ACR SAMs, piranha-treated glass, and powdered ASA crystallized in bulk. Vertical lines refer to reference literature values for bulk ASA reflections. Marked in red are the reflections detected in thin-film crystallized samples with the respective Miller indices. (b) Ratio between the intensity of reflections from (100) and (002) + (011) planes of ASA thin films crystallized on different substrates and corresponding acid surface tension component. Error bars refer to standard deviations. SEM images of ASA thin films crystallized via spin coating on (c) ACR and (d) GLY SAMs. The scale bar is 1 μm. Diagrams in the inset show the relationship between film morphology and crystal orientation on the substrate.

After ensuring control over thin film attributes and reproducibility, SCC of ASA was carried out on other SAMs. The different interactions occurring at the surface–solution interface were investigated by analyzing the crystallographic features of the films. First, powdered ASA crystals (bulk form) were analyzed by XRD to identify the reflections of unconstrained crystallization. Diffraction pattern and assignments^50,51^ are reported in Figure S5. Many reflection planes could be detected because of the absence of dimensional constraints in bulk crystal growth. Note that the peak at 15.4° resulted from the convolution of (002) and (011) crystal planes, which were thus considered together. XRD characterization of the thin ASA films grown on bare glass and SAMs-grafted substrates is presented in Figure 4a. Comparing the diffraction patterns of thin films grown on glass substrates with those of ASA bulk crystals, it was evident that SCC itself had a strong effect on ASA crystallization. It is known that high supersaturation rates, such as those achieved under fast solvent evaporation conditions, can promote the nucleation of different crystal polymorphs.^52^ Nonetheless, the accelerated kinetics of nucleation promoted by SCC did not alter the ASA crystal form since XRD confirmed that thin films made of monoclinic crystals were always obtained. Thus, the crystal form was not influenced by the crystallization technique. In all the ASA thin films, only peaks relative to (100), (002) + (011), (112), and (022) crystalline planes were detected. The appearance of fewer crystalline planes compared to the spectra of powdered ASA can be attributed to the dimensional constraints over crystalline growth imposed by the limited thickness of the spin-coated film. When SCC was carried out on SAMs, ASA constrained growth along the same crystalline planes identified on bare glass was observed, but the intensity ratio between the peaks was different (see Figure S6). Thus, the relative intensity of reflections corresponding to different crystal orientations was not determined by SCC but by the SAM surface chemistry.

Figure 4b reports the ratio between (100) and the convolution of (002) and (011) planes of ASA crystals grown in bulk and as a thin film. ASA crystal faces are characterized by different polarity and acidity according to the exposed groups.^51,53^ Generally, it can be seen that SCC reduced (002) + (011) while enhancing (100) reflections and that the minimum value of the ratio was obtained for powdered ASA grown in the bulk. SCC on piranha cleaned glass surfaces was found to slightly increase such a ratio, but the major role was played by the insertion of specific surface chemistries via SAM grafting. ACR SAMs led to the ratio closest to 1, pointing out that functionalization with methacrylate groups favored the growth of crystal planes exposing both donor and acceptor groups. In a specular way, methacrylate end groups expose both donor- and acceptor-type terminations. The interactions between the surface and ASA carboxyl groups, favoring the growth of (011) acceptor plane, and ASA benzene rings, favoring donor planes, resulted in an increase of exposed (100) facets with respect to the bulk and bare glass case. Conversely, THIOL, AMINO, and GLY SAMs led to ratios between 4 and 4.5, indicating a strong predominance of planes displaying electron donor features within the ASA thin film. For example, thiol groups preferentially interacted via hydrogen bonding with ASA carbonyl groups, thus greatly enhancing the growth of (100) crystal planes. These conclusions were further supported by the strong correlation observed between the acid component of SAM surface free energy (γ^+^, see Table 2) and the (100)/[(002) + (011)] intensity ratio (Pearson’s *r* = −0.964). As highlighted in Figure 4b, low γ^+^ surfaces (THIOL, AMINO, and GLY SAMs) determined stronger interactions with donor ASA crystal facets and thus preferentially grew (100) carbonyl terminated planes, while high γ^+^ surfaces (piranha-treated glass and ACR SAMs) interacting preferentially with acceptor facets favored the growth of (002)+(011) crystal features. Assuming that nucleation and crystallites formation during solvent evaporation in SCC proceeds governed by interfacial tensions between individual facets and SAM surface, solvent type, and evaporation time (which are the same for all the substrates), one could look at the variation of the intensity ratios between different crystal facets as a consequence of their preferential interaction with SAM surface chemistry and the resulting orientation of the ASA crystallites with respect to the surface normal. This interpretation is supported by the observed morphology of ASA SC-crystallized thin films in cross-sectional SEM images of Figure 4c,d. Additional SEM images are reported in Figure S7. Controlling and enhancing preferential growth is important since ASA (100) crystal face displays mild hydrophilicity compared to the other ASA faces, thus being rather water soluble. Its extended growth is highly desirable in the frame of efficient and fast drug administration.^35^

### 3.3 Direct Evaluation of ASA (100) Plane–Functional Group Interactions

The crystallization of ASA in thin films highlighted the preferential growth of certain crystal facets imposed by SAM surface chemistry. As a step further, the molecular interaction between ASA and functional groups was investigated at a higher degree of detail by AFM force spectroscopy. A similar approach has been employed to investigate surface–protein interactions^54^ and for chemical sensing experiments.^55^ This technique represents a powerful tool for testing the affinity between the heteronucleant surface chemistry and the solute, but the tip functionalization can be a tedious process since extreme care must be taken during handling to avoid the detachment of the probe. In addition, the ASA crystals must show an extended and defect-free area of the target crystal facet and must not move or fall on the AFM stage during the measurement.

**Figure 5:**
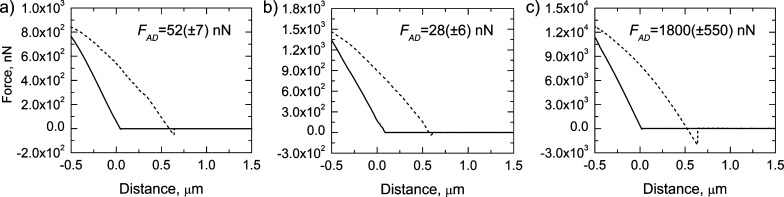
Force–distance curves between the (100) ASA crystal face and the (a) unfunctionalized AFM tip, tip functionalized with (b) methacrylate and (c) thiol groups. The continuous line refers to the snap-in phase, whereas the dashed line refers to the snap-back phase. The adhesion force values are reported in the insight.

AFM tips were functionalized with silanes, and the adhesive force when facing a (100) ASA crystal plane was measured, as schematized in Figure S8. In this way, the interaction between functional groups and a specific crystal plane could be quantified in the absence of solvents and eventually correlated with the crystallization outcome. Force–distance curves involving unfunctionalized tips and tips carrying methacrylate and thiol groups are reported in Figure 5. When the tip carrying methacrylate groups was considered, the adhesive force, *F*_AD_, between the ASA crystal and the tip was very low, namely, 28 (±6) nN. The weak interaction between methacrylate groups and ASA well agreed with the orientational considerations made for ASA thin films, as the growth of the (100) face was inhibited compared to other SAMs. On the other hand, considering tips carrying thiol groups, the adhesion force dramatically increased, being as high as 1800 (±550) nN. An intense hysteresis was also observed. During the approaching phase, or snap-in, long-range interactions prevailed, and a strong electrostatic affinity was identified between thiol groups and the ASA (100) plane. The strong interaction resulted in a marked discontinuity in the force values during the snap-out phase and high adhesive force. Such a result agreed with the large negative values of SZP of THIOL SAMs and the hypothesized mechanism of interaction for ASA nucleation. In addition, the evaluation of force–distance curves required much stiffer tips, when functionalization with thiol groups was considered, to successfully withdraw the tip from the crystal surface and avoid the detachment of the cantilever. All the evidence further corroborated the diversified preferential interactions between SAMs and ASA crystal planes.

## 4 Conclusions

The influence of SAMs on the crystallization of a model API molecule, namely, ASA, has been discussed. SAMs guaranteed fine functionalization coupled to very low roughness to deconvolute the influence of surface chemistry on API nucleation from morphological effects, as much as physically possible. SAMs effectively tuned ASA nucleation kinetics during batch crystallization, being either nucleation promoters or inhibitors according to their surface hydrophobicity. To evaluate the surface–API interaction in a rapid and money-effective way, ASA was also crystallized on SAMs in thin films. The face-selective action of SAMs resulted in different relative growths of crystal planes of ASA, as highlighted by XRD and SEM analyses, and was related to the different molecular interactions of the SAM end groups with specific moieties exposed by ASA molecules on crystal facets. The crystallization outcomes were related to the acid surface tension component of SAMs and, in particular, to the matching between the donor/acceptor features of the surface and the crystal plane. The diversified interaction was also confirmed by the direct evaluation of the adhesive force between the crystal plane and SAM end groups. Overall, crystallization in thin films has the potential to serve as a tool to help drug design and discovery. In this first study, we studied crystallization on various substrates without changing the solvent of the mother liquor. The interaction of the API with the solvent is another key point in pharmaceutical crystallization and will be addressed by SCC in future studies. Future developments will also involve the application of SAMs to the tuning of the crystallization of biopharmaceuticals.

## Supporting Information Available

Additional details on the experimental section and data regarding the physicochemical characterization of SAMs and ASA thin films (PDF)

## Abbreviations

ACR: 3-(trimethoxysilyl)propyl methacrylate
AFM: atomic force microscopy
AMINO: 3-aminopropyltrimethoxysilane
API: active pharmaceutical ingredient
ASA: aspirin
FE: field emission
GLY: 3-glycidyloxypropyltrimethoxysilane
HB: hydrogen bond
SAM: self-assembled monolayer
SCC: spin-coating crystallization
SEM: scanning electron microscopy
SZP: surface zeta potential
THIOL: 3-mercaptopropyltrimethoxysilane
TLD: through-lens detector
USP: United States Pharmacopoeia
XRD: X-ray diffractometry

## Nomenclature

γ: surface tension
γ^AB^: polar component of surface tension
γ^+^: acid component of surface tension
γ^–^: basic component of surface tension
γ^LW^: Lifshitz–van der Waals component of surface tension
*F*_AD_: adhesive force
*S*: supersaturation
τ: nucleation induction time
*t*: time
*t*_lag_: nucleation lag time
*t*_nuc_: overall nucleation induction time
ω: rotational speed
