# Supplementary material for: Role of Self-Assembled Surface Functionalization on Nucleation Kinetics and Oriented Crystallization of a Small-Molecule Drug: Batch and Thin-Film Growth of Aspirin as a Case Study
Source: ACS Appl Mater Interfaces. 2021 Mar 24;13(13):15847–56. doi: 10.1021/acsami.1c00460 (PMC8041258; doi:10.1021/acsami.1c00460)
Supplement: Supplementary file 1 — am1c00460_si_001.pdf [file am1c00460_si_001.pdf]

SUPPORTING INFORMATION:

Role of self-assembled surface functionalization on nucleation kinetics and oriented crystallization of a small-molecule drug: batch and thin-film growth of aspirin as a case study

Fiora Artusio[1], Francesco Fumagalli[2], Andrea Valsesia[2], Giacomo Ceccone[2], Roberto Pisano[1*]*

[1] Department of Applied Science and Technology, Politecnico di Torino, corso Duca degli Abruzzi 24, 10129, Torino, Italy

[2] European Commission, Joint Research Centre (JRC), via E. Fermi 2749, 2102, Ispra, Italy

*Corresponding authors, e-mail: roberto.pisano@polito.it, Tel. +39 011 0904679

e-mail: fiora.artusio@polito.it, Tel. +39 011 0904694

1. MATERIALS AND METHODS SUPPORTING INFORMATION

1.1 Materials

Borosilicate glass coverslips were purchased from Neuvitro (diameter = 14 mm, D263M, Vancouver, USA). Hydrogen peroxide (30 wt% in water, ACS reagent), sulfuric acid (ACS reagent, 95.0 – 98.9%), anhydrous toluene (<0.001% water, 99.8%), toluene (ACS reagent, ≥ 99.5%), 3-aminopropyltrimethoxysilane (AMINO, 97%), 3-glycidyloxypropyltrimethoxysilane (GLY, ≥ 98%), 3-mercaptopropyltrimethoxysilane (THIOL, 95%) and 3-(trimethoxysilyl)propyl methacrylate (ACR, 98%) were used for SAM synthesis. Aspirin (ASA, USP grade), ethanol (puriss. p.a., absolute, ≥ 99.8%), and deionized water were used for crystallization trials. All the reagents were purchased from Sigma-Aldrich (Cesano Maderno, Italy).

1.2 Synthesis of SAMs on glass

Glass coverslips were pre-activated by immersion in 3:1 piranha solution for 1 h. Substrates were thoroughly rinsed with water, blown dry with nitrogen, and immediately transferred into 0.054 M silane solutions in anhydrous toluene. Reaction time was set at 15 h for all the silanes, except for AMINO. In this case, 30 min were enough for reaching a good-quality SAM. Then, coverslips were generously rinsed with toluene, toluene/ethanol (1:1), and ethanol. All the surfaces were freshly prepared before each crystallization trial. The detailed and optimized synthesis is described in our previous study[1].

1.3 Physico-chemical characterization of SAMs

In the present study, SAMs were mainly characterized in terms of surface tension and topography. The detailed characterization is reported elsewhere[1]. Three probing liquids were used to carry out contact angle analyses (MSE DigiDrop, GBX, France). Two polar, i.e., water and glycerol, and one nonpolar, i.e., diiodomethane, liquids were selected. 3 µL drops were dispensed over the functionalized glass surface. The van Oss-Chaudhury-Good (vOCG) model was applied to calculate the dispersive, acid, and base surface tension components:

$$\gamma_l(1 + cos\theta) = 2\left[\sqrt{\gamma_l^{LW}\gamma_s^{LW}} + \sqrt{\gamma_l^{+}\gamma_s^{-}} + \sqrt{\gamma_l^{-}\gamma_s^{+}}\right] \quad (1)$$

where l and s subscripts refer to liquid and solid components of surface tension. γ^{LW}, γ^{+}, and γ^{-} refer to the Lifshitz-Van der Waals, acid, and base components of surface tension. θ is the contact angle between the surface-liquid pairs.

Surface topography was evaluated with atomic force microscopy (AFM, Solver NANO, NT-MDT Spectrum Instruments, Russia). The instrument was operated in tapping mode. Si_3N_4 tips were used, the cantilever frequency was set at 0.8 Hz, and the tip curvature radius was 10 nm. A 1x1 μm^2 area was analyzed, and each scan consisted of 256 lines. Images were processed on Gwyddion software (ver. 2.51, Czech Metrology Institute) with a 5th order polynomial. To calculate RMS roughness, analyses were performed in triplicate, and three measurements per surface were carried out.

The effective chemical functionalization of glass was explored by X-ray photoelectron spectroscopy (XPS) (AXIS ULTRA, DLD Kratos Analytical, UK) equipped with a monochromatic Al Kα source (hν = 1486.6 eV) operating at 150W. Wide scan spectra were recorded from 1100 to 0 eV binding energy in hybrid mode, “slot” aperture (400 × 700 μm^2 analysis area), and at 160 eV pass energy. The take-off angle (ToA), respect to the sample normal, was 0°. The operating pressure was 6×10^{-7} Pa. Surface charging was compensated using low energy (~5 eV) electrons and adjusted using the charge balance plate on the instrument. Three different spots were analyzed for each sample. All the spectra were processed with CasaXPS (ver. 2.3.20). Spectra were calibrated setting hydrocarbon C1s at 285.0 eV. The surface composition was evaluated from the survey spectra, after a Tougaard U3-type background subtraction, using the relative sensitivity factors provided by the manufacturer.

The thin film thickness was evaluated by ellipsometry techniques (alpha-SE, J.A. Woollam Co., Lincoln, NE, USA). For all the measurements, 65°, 70°, and 75° angles between the incident light source and detector were used. First, a model describing the intensity of light as a function of the wavenumber has been developed for the untreated substrate using COMPLETEEASE software (ver. 4.63, J.A. Woollam Co.). Knowing the refractive index (1.523), the thickness (0.15 mm), and the roughness (0.128 nm) of coverslips, a Cauchy model has been implemented, and parameters such as the complex refractive indexes and the angle offsets for untreated glass were calculated by fitting experimental spectra. Then, for functionalized samples, the layer thickness was calculated by setting the defined model for bare glass and fitting the refractive index and thickness of the additional Cauchy over layer. In every case, mean square error (MSE) was below 2.5.

Measurements were carried out in triplicate and on three different spots per sample, in order to test sample and intra-batch uniformity.

1.4 Batch crystallization and kinetics evaluation

The nucleation kinetics of ASA was evaluated by means of a specifically designed *in-house* equipment. ASA was first dissolved in an ethanol/water mixture (38/62 v/v) and then passed through PTFE syringe filters with 0.45 and 0.22 µm pore size (Millex, Millipore). Starting concentration was 31.6 mg/mL to guarantee a supersaturation, *S*, of 1.8 at the crystallization temperature, T_c. *S* was defined as:

$$S = \frac{C}{C_s} \tag{2}$$

where *C* is the actual ASA concentration and C_s is the ASA solubility at a given temperature. T_c was set at 15 °C to maximize the probability of observing heterogeneous rather than homogeneous nucleation. In this way, the effect of SAMs chemistry on nucleation could have been studied.

SAMs were placed at the bottom of each well of 24-well plates. Then, the wells were filled with 125 µL of ASA solution using an automatic pipette (M4 Multipette, Eppendorf, Germany) to minimize solvent evaporation during dispensing. Plates were covered with a lid to isolate the crystallization environment and avoid contamination. All the operations were carried out inside a laminar flow hood (Asalair 1500, Asal, Milan, Italy) to minimize the presence of impurities inside the system. Particles or other contaminants are likely to act as hazardous heteronucleants, thus altering the nucleation kinetics and preventing the correct identification of the impact of SAMs on nucleation. Two plates were placed inside a chamber completed with a cooling system. A proportional temperature controller has been designed on LabVIEW and guaranteed a temperature stability of ± 0.1 °C throughout the whole duration of the experiment. A thermocouple (type T) was inserted inside a well filled with ASA-free ethanol/water mixture to monitor the temperature of the liquid. Dry nitrogen was fluxed inside the chamber, and the controller constantly regulated the "hot" and "cold" flows to ensure equilibrium between the inlet thermal flux and the outlet flux due to the thermal dispersion towards the environment. The chamber was designed to inspect the wells *via* time-lapse optical microscopy, as the top and the bottom walls were made of optically transparent glass. The thermally insulated chamber was placed under a stereomicroscope coupled to a motorized X-Y stage

(M125C, Leica, Germany). A map of positions was identified and implemented on LAS-X software (Leica, Germany), and time-lapse images of 47 samples were automatically acquired every 2 h at 1X in transmitted light mode.

Data acquired with time-lapse monitoring were then used to calculate the cumulative probability of observing nucleation events in the ensemble of wells. All the curves were referred to the same number of independent samples for internal data consistency. Poisson's statistics was fitted to the experimental data to calculate the nucleation induction time, τ. The general expression of Poisson's law is

$$f(\nu, n) = \frac{\nu^n e^{-\nu}}{n!} \tag{3}$$

where *ν* and *n* are the expected and the exact number of events in a defined interval, respectively. *n* was set at 0 and *ν* at - t/τ to obtain the probability of not encountering nucleation events within time:

$$f\left(-\frac{t}{\tau}, 0\right) = P(t) = e^{-\frac{t}{\tau}} \tag{4}$$

from which τ could have been calculated. The Poisson's law is widely applied to describe nucleation kinetics, and was demonstrated to well describe crystallization phenomena, provided that crystal growth proceeded much faster than nucleation, and that supersaturation was rapidly achieved.

1.5 Thin film crystallization

ASA thin film crystallization was achieved by using a spin coater (Apogee, Cost Effective Equipment, Missouri, USA). ASA was dissolved in pristine ethanol to obtain 50 mg/ml solutions. As for batch crystallization, solutions were passed through 0.45 and 0.22 µm syringe-filters. Bare or SAM-functionalized coverslips were placed onto the chuck and vacuum down to 20 Pa was applied to keep the sample in place. 100 µL of ASA solution were pipetted onto the coverslip. Acceleration at 500 rpm/s followed immediately after the dispensing step, and stationary rotational speed varied between 500 and 4000 rpm. In this way, the excess of solution was removed, and solution thickness was controlled after reaching the target rotational speed. Then, the solvent progressively evaporated, approaching supersaturation conditions. When enough driving force was achieved, nucleation was initiated, followed by fast crystallization. Spin time was 5 min to

ensure complete solvent removal. At the end of the process, the sample was recovered and ready for XRD analyses.

1.6 Ellipsometry

Ellipsometry was employed to measure the ASA film thickness. Measurements were performed at 65°, 70°, and 75° to build a proper mathematical model describing the system (alpha-SE, J.A. Woollam Co., Lincoln, NE, USA). First, models for untreated glass and SAM-functionalized glass were developed. The refractive indexes and thickness of different SAMs were calculated by fitting the trend of light intensity to a Cauchy model. Then, the thickness of ASA thin film crystallized on SAMs was calculated.

1.7 Field Emission Scanning Electron Microscopy (FE-SEM)

FE-SEM (FEI NOVA 600 Dual Beam, Hillsboro, OR, USA) was selected to investigate the morphology of ASA thin films. Samples were carefully cleaved to allow the imaging of the film cross-section and metallized with a thin layer of gold to avoid surface charging. The FE-SEM accelerating voltage was set at 2 keV, a through-lens detector (TLD) was used, and the working distance was set at 5 mm.

1.8 X-Ray Diffraction (XRD)

ASA thin films were analyzed with no preliminary treatments with an X-ray diffractometer operated in Bragg-Brentano mode (D8 Advance, Bruker AXC GmbH, Karlsruhe, Germany). A 0D-LYNXEYE detector was selected to carry out the measurements. Current and voltage were set at 40 mA and 40 kV, respectively. A Göbel mirror, 2.5° soller, and a 0.3 mm pinhole were inserted along the primary beam path. A notch knife was used to avoid saturating the detector counts at small angles. A 0.6 mm slit and 2.5° soller were mounted on the secondary beam path. In a typical scan, 2θ ranged from 6 to 35°, step size was 0.02°, and time per step was 15 s.

1.9 Force spectroscopy and tip functionalization

The interaction between ASA (100) crystal face and selected SAM chemistries was evaluated with AFM (Solver NANO, NT-MDT Spectrum Instruments, Russia) by means of force spectroscopy. SiN_4 tips were functionalized with silanes following a similar procedure as for glasses. Extreme care was taken throughout the whole functionalization process to avoid damaging or breaking the tip. In this perspective, aggressive piranha pretreatments were substituted with more gentle exposures to UV to activate the surface. Tips were firstly hydroxylated by exposure to UV for 15 min. Immediate incubation in silane solution followed to avoid the decay of the labile surface plasma. Tips were immersed in 0.054 M solutions of MPTMS or TMSPM dissolved in anhydrous toluene for 15 h. Tips were carefully rinsed with the same sequence of solvents used for SAM synthesis on glass and dried with nitrogen. The functionalized tips were then mounted on the AFM piezo and used to collect force-distance curves. Each probe was preliminarily calibrated using silicon wafers, and spring constant was calculated. A UV-cleaned tip was taken as reference. Force-distance curves were measured using an ASA crystal grown in bulk using batch crystallization. The crystal was placed on the AFM stage with the extended (100) face facing the tip. Each measurement was repeated at least on 15 different spots of the ASA crystal surface. When the attractive force exceeded the cantilever mechanical resistance, a detachment of the tip was observed. Such a phenomenon occurred for THIOL functionalized tips, and tips were substituted by stiffer ones. Results were collected using NOVA software (NT-MDT, Russia). The force constant of the cantilever, k, was measured using the Sadler method and used for the calculation of the tip-surface interaction force F_{AD} ($F_{AD} = -kd$, where d is the deflection of the cantilever). Force constants were 1.5, 1.8, and 22.0 N/m for UV-cleaned, ACR, and THIOL functionalized tips, respectively.

2. RESULTS SUPPORTING INFORMATION

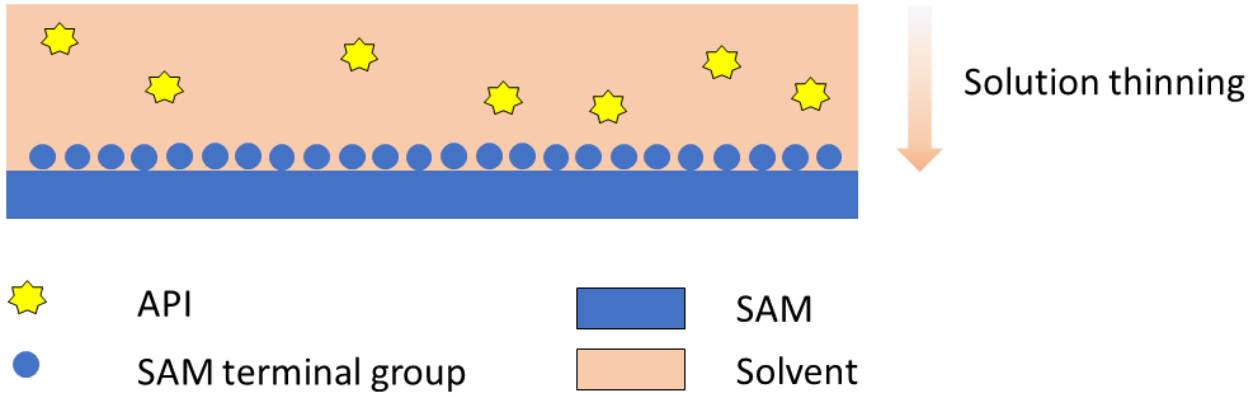

Figure S1. The isotropy of interfacial interactions between API solute molecules and SAM exposed groups during the spin-coating process.

Table S1. Contact angle values of water, glycerol and diiodomethane measured on activated glass and SAMs exposing different groups[1]. Standard deviations are reported in brackets.

	θ_{water}	$\theta_{glycerol}$	$\theta_{diiodomethane}$
**ACTIVATED GLASS**	10 (± 1.8)	8.5 (± 1.0)	42.0 (± 0.8)
**THIOL SAM**	65.7 (± 1.7)	57.3 (± 0.9)	46.4 (± 0.8)
**AMINO SAM**	33.8 (± 2.6)	34.7 (± 0.6)	32.5 (± 0.2)
**ACR SAM**	65.4 (± 1.7)	47.7 (± 0.9)	41.9 (± 0.7)
**GLY SAM**	62.8 (± 1.5)	54.2 (± 1.2)	44.5 (± 0.9)

Table S2. Atomic composition of activated glass and SAMs exposing different groups as measured with XPS[1]. Standard deviations are reported in brackets.

	O%	Si%	C%	S%	N%	Rest.% (K, Al, Na, Zn, Ti)
**ACTIVATED GLASS**	64.47 (± 0.37)	25.08 (± 0.23)	8.13 (± 0.71)	-	-	< 3
**THIOL SAM**	55.73 (± 0.63)	21.43 (± 0.19)	17.65 (± 0.79)	2.98 (± 0.14)	-	< 3
**AMINO SAM**	55.02 (± 0.37)	19.40 (± 1.00)	21.32 (± 1.20)	-	4.11 (± 0.06)	
**ACR SAM**	59.83 (± 0.51)	20.76 (± 0.17)	16.09 (± 0.56)	-	-	< 3
**GLY SAM**	53.85 (± 0.10)	18.68 (± 0.37)	25.43 (± 0.21)	-	-	< 3

Table S3. Thickness and packing factors, f_{mol}, of SAMs exposing different groups as measured by ellipsometry[1].

	**Thickness, nm**	**f_{mol}, %**
**THIOL SAM**	0.75	99.8
**AMINO SAM**	0.76	100
**ACR SAM**	0.83	81.2
**GLY SAM**	0.90	98.6

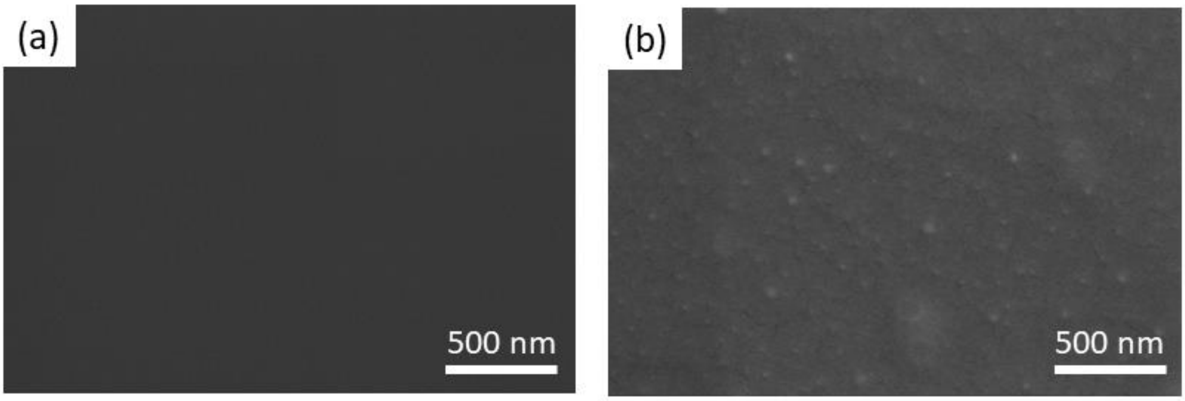

Figure S2. SEM micrographs of (a) untreated glass and (b) SAM exposing thiol groups. The immobilization of thiol groups on the glass surface did not alter the glass morphology[1].

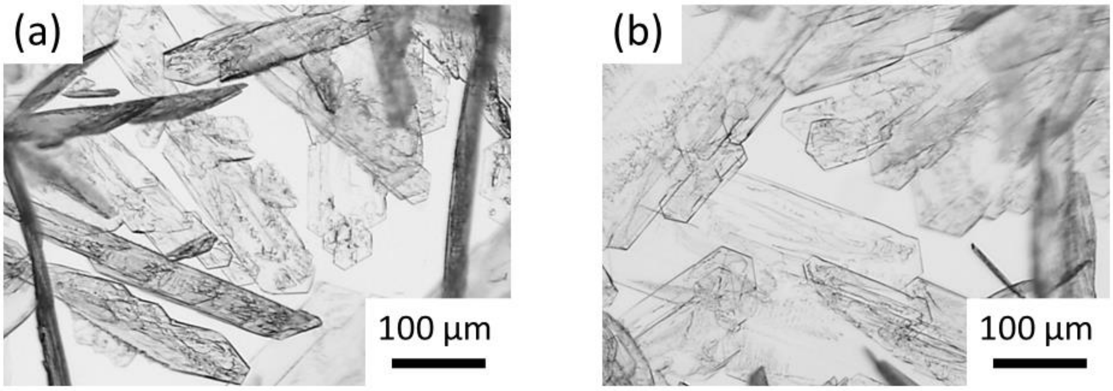

Figure S3. Representative optical microscope images of ASA crystals grown via batch crystallization on a) AMINO and b) GLY SAMs functionalized surfaces.

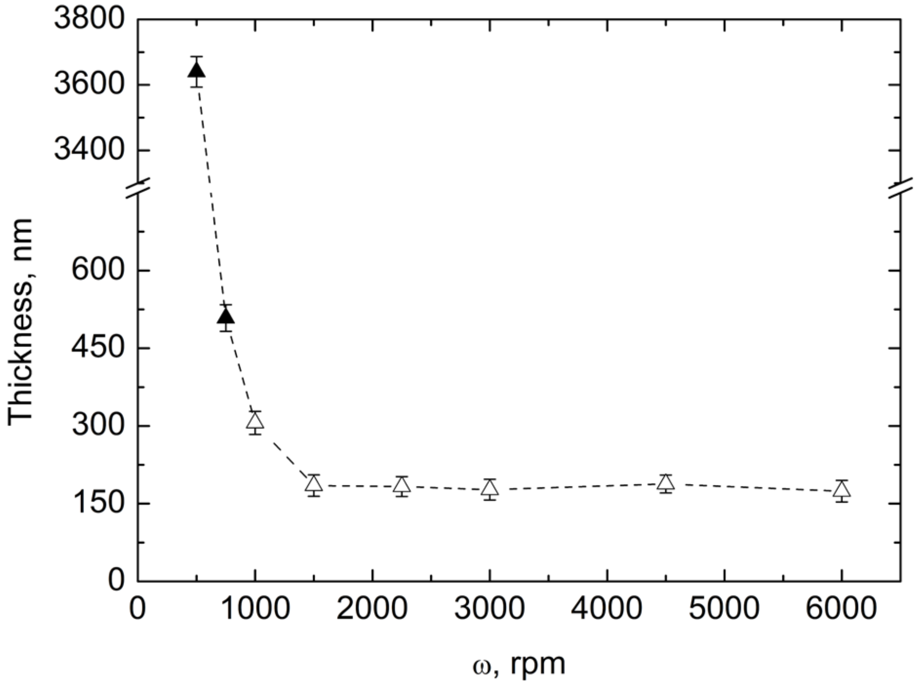

Figure S4. Thickness of ASA thin films grown on THIOL SAMs as a function of the spin coating rotational speed. Results were obtained by ellipsometry for $\omega \geq 1000$ rpm and by FE-SEM evaluation of thicker samples' cross-section for $\omega < 1000$ rpm. The amount of crystalline material on the SAM surface hindered the beam location by the ellipsometer detector for films thicker than few hundred nanometers. Full symbols refer to values obtained by FE-SEM, whereas open symbols refer to ellipsometry. Error bars refer to standard deviation. The smaller standard deviations obtained at high ω mirrored the fine tuning of film thickness.

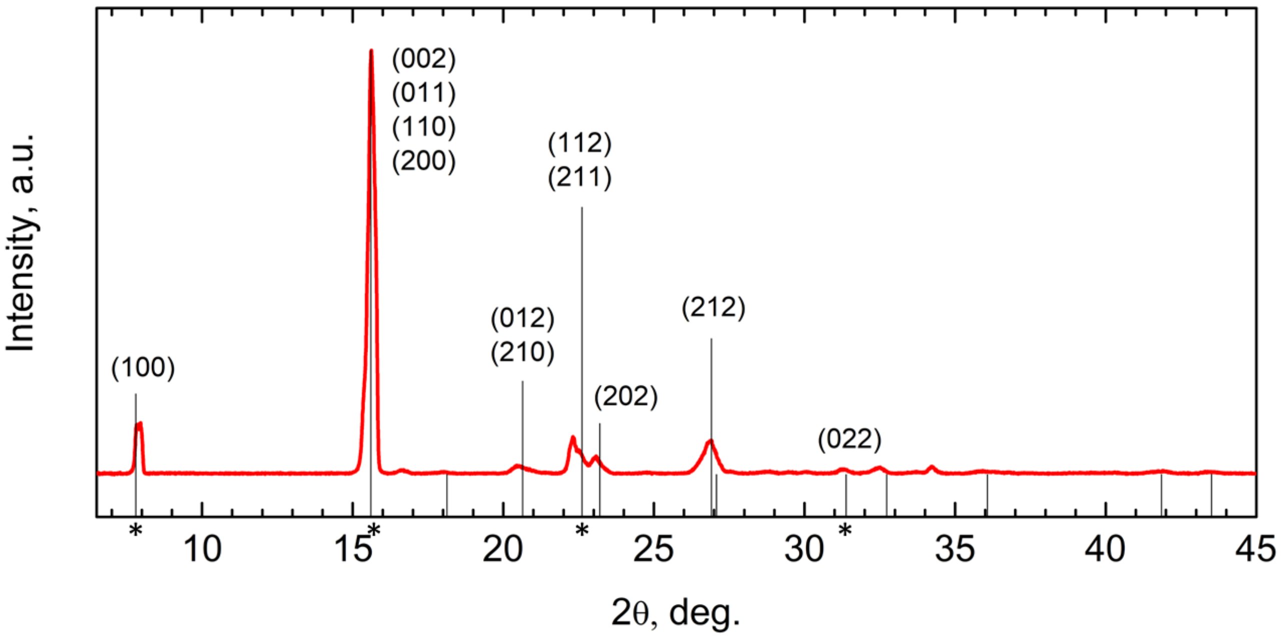

Figure S5. XRD diffraction pattern of powdered reference ASA crystals (bulk). Black lines show reference reflections position and relative intensities with associated Miller indices. Asterisks denote reflections also observed in spin coated ASA thin films.

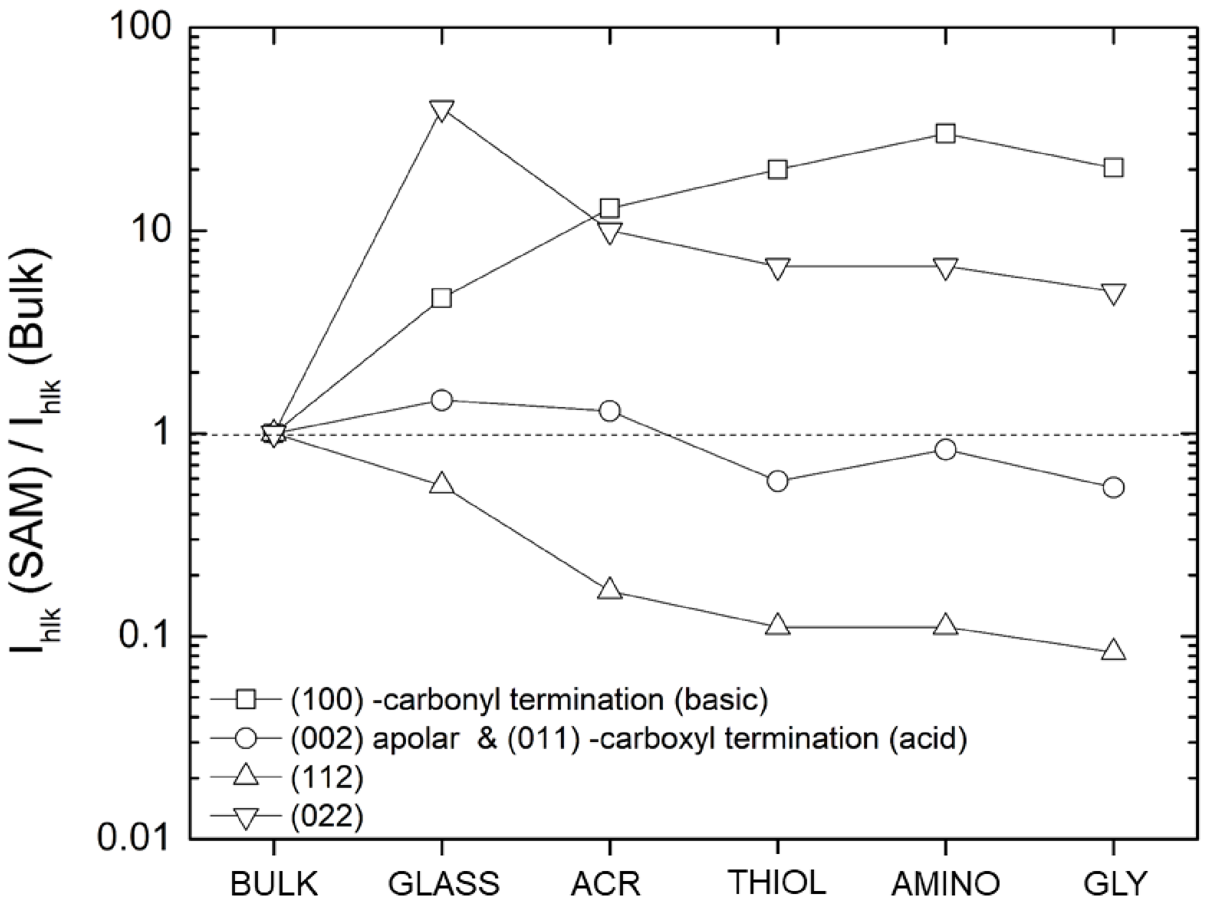

Figure S6. XRD reflections intensities (normalized to powdered reference ASA crystals intensities) for reflections observed in ASA thin films.

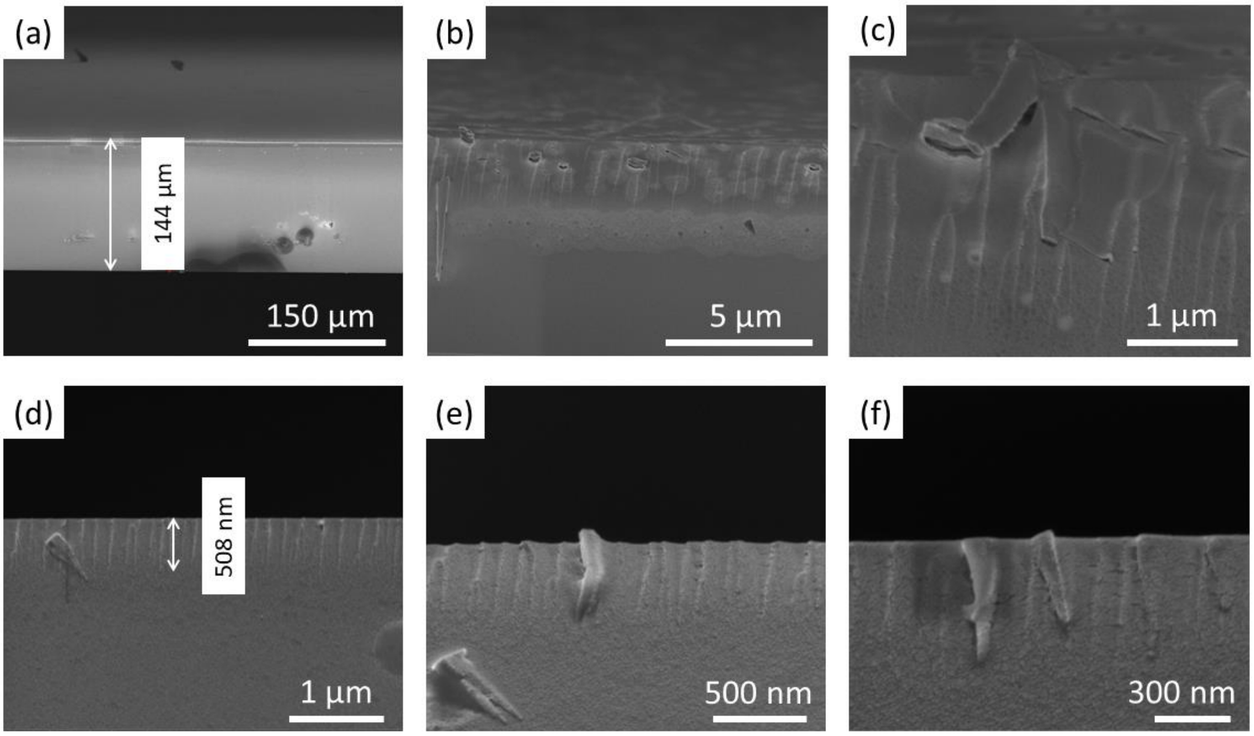

Figure S7. SEM images showing the morphology of spin coated ASA films on ACR SAMs (thickness 500nm) at different magnifications. Panel a) shows the thickness of the supporting glass slides.

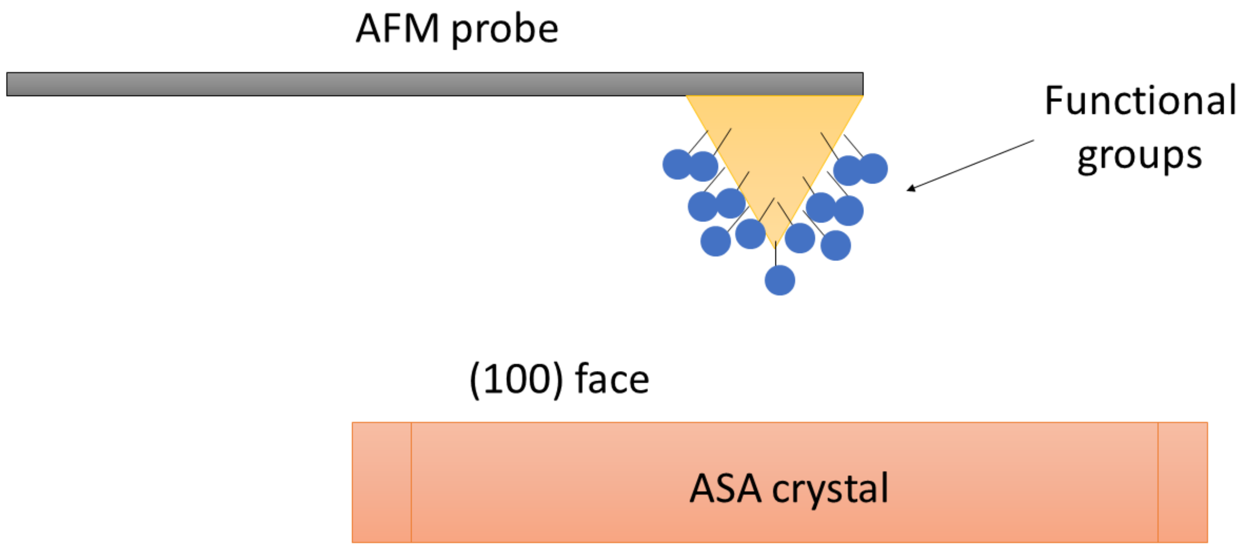

Figure S8. Schematic representation of the experimental set-up developed to study ASA – functional groups interactions via AFM force spectroscopy.

REFERENCES

(1) Artusio, F., Fumagalli, F., Bañuls-Ciscar, J., Ceccone, G., Pisano, R. General and adaptive synthesis protocol for monolayers as tunable surface chemistry platforms for biochemical applications. *Biointerphases* **2020**, *15* (4), 041005. https://doi.org/10.1116/6.0000250.
